# Treatment of Indolent Ulcers—Particularly Those of a Lyphititic Character

**Published:** 1860-03

**Authors:** B. Woodward

**Affiliations:** Galesburg, Ill.


### ARTICLE 4.

### TREATMENT OF INDOLENT ULCERS.

### PARTICULARLY THOSE OF A LYPHITITIC CHARACTER.

BY B. WOODWARD, M. D., OF GALESBURG, ILL.

The *Chicago Medical Journal* for the present month, contains an editorial on "the treatment of indolent ulcers by vapor of iodine," and the mode of using the vapor. During the winter of 1856–7 it was my privilege to attend the teachings of Professor Brainard at the Marine Hospital, Chicago; and while there, had the opportunity of watching this mode of treatment of various cases in the hospital. Of several of these cases I kept copious notes, some of which I will condense for the present article.

Morris Joice, seaman, on admission to the hospital, November 3, 1855, had an ulcer of eight months standing, covering the middle of tibia of the right leg. The ulcer was five inches long, and three inches broad; surface much depressed, and covered with gray exudation; outline regular, and edges ragged. Professor B. directed it to be treated by iodine vapor. No constitutional treatment, as he wished to test the iodine treatment.

On the 12th of the same month, the ulcer had become reduced in size, one half, covered with coagulated lymph, and healthy granulations all over the surface.

November 22d. Ulcer not larger than a twenty-five cent piece; granulations rise even with surrounding surface; continue vapor of iodine. By the 8th of December, the leg was entirely healed. This man had never had syphilis.

November 14th. Thomas Rigby, seaman, admitted to hospital, with a large ulcer over the external malleolus, of several weeks standing. Ulcer an inch and three quarters long, and an inch and a quarter wide; surface much depressed, and covered with a gray exudation mixed with coagulated blood.

Recipe—Iod. potassa, five grains in solution, three times a day, and to ulcer iodine vapor. This treatment was continued ten days, by which time the ulcer had become reduced to the size of a five cent piece. By the 5th of December the ulcer was entirely healed.

During the winter the treatment by vapor of iodine, was adopted in the hospital in several other cases, with the same marked success.

The doctrine of Professor Brainard was, that an ulcer was an absorbing surface, and that iodine applied in this way had not only a local, but a constitutional effect. To prove this, at the request of the Professor, Drs. Powell, Durham, and myself, subjected the urine of Morris Joice and two others, on whom iodine had been used in no other way than the local application of the vapor, to chemical test, and in each case the presence of the iodine in the urine was proven.

Since that time I have used iodine by Dr. Brainard's method in the treatment of indolent ulcers, with the best results. Where the ulceration has been the result of syphilis, I have found the exhibition of iodide of potassa to materially aid in effecting a cure. In one case of scrofulous ulcer of the neck in a young woman, which had remained open for four months, the iodine vapor effected a cure in three weeks. I am now using it in a bad case of ulceration of the foot from frost bite, with the best prospects of success.

